# Legionnaires’ Disease at a Dutch Flower Show: Prognostic Factors and Impact of Therapy

**DOI:** 10.3201/eid0812.020035

**Published:** 2002-12

**Authors:** Kamilla D. Lettinga, Annelies Verbon, Gerrit-Jan Weverling, Joop F.P. Schellekens, Jeroen W. Den Boer, Ed P.F. Yzerman, Jacobus Prins, Wim G. Boersma, Ruud J. van Ketel, Jan M. Prins, Peter Speelman

**Affiliations:** * Academic Medical Center, Amsterdam, the Netherlands; † National Institute of Public Health and the Environment, Bilthoven, the Netherlands; ‡ Municipal Health Service Zuid-Kennemerland, Haarlem, the Netherlands; § Regional Laboratory of Public Health Haarlem, Haarlem, the Netherlands; ¶ Westfries Gasthuis, Hoorn, the Netherlands; # Medical Center Alkmaar, Alkmaar, the Netherlands

**Keywords:** Community acquired infections, Legionella pneumophila, Legionnaires’ disease, intensive care units, antibiotics, prognosis, risk factors

## Abstract

After a large outbreak of Legionnaires’ disease in the Netherlands, we determined risk factors for intensive care unit (ICU) admission and death and the impact of adequate therapy on ICU-free survival among 141 hospitalized patients. Overall mortality rate was 13%, and ICU mortality rate was 36%. Smoking, temperature >38.5°C, and bilateral infiltrates shown on chest x-ray were independent risk factors for ICU admission or death (all p<0.05). Starting adequate therapy within 24 hours after admission resulted in a higher ICU-free survival rate compared to therapy initiation after 24 hours: 78% versus 54%, respectively (p=0.005). However, delay in providing therapy to patients with urinary antigen tests with negative results did not influence outcome. These data suggest that by using the urinary antigen test on admission a more tailored approach to patients with community-acquired pneumonia may be applied.

 Severe Legionnaires’ disease has an overall mortality rate of 10% to 30% (1–3), and 30% to 50% of patients require admission to an intensive care unit (ICU) (1,4). One of the most important determinants of outcome is the early initiation of adequate therapy after admission (1,5). Administering appropriate antibiotics for Legionella pneumophila during the empiric treatment of patients with community acquired pneumonia has been advocated (6). Given the low frequency of Legionnaires’ disease, this strategy is costly and leads to overconsumption of antibiotics. Therefore, many physicians have not adopted these guidelines in daily practice. Identifying those patients with community-acquired pneumonia caused by L. pneumophila is difficult.

In March 1999 one of the largest outbreaks of Legionnaires’ disease since the first described outbreak in Philadelphia (7) occurred in the Netherlands. The outbreak originated at the Westfriesian Flora, an annual flower show combined with a consumer products exhibition, held February 19–February 28, 1999. The flower show was visited by 77,061 persons, and Legionnaires’ disease developed in at least 188 (8). The size of the outbreak provided a unique opportunity to determine which clinical factors on hospital admission predict ICU admission or death (ICU/death). We also evaluated whether the rapid urinary antigen test can help identify those patients with Legionnaires’ disease for whom adequate therapy cannot be delayed.

## Patients and Methods

### Study Group

On March 12, 1999, the Dutch population was alerted by newspapers and a special broadcast that a flower show was identified as probable origin of an outbreak of Legionnaires’ disease (9). To collect clinical data, we obtained written informed consent from patients with Legionnaires’ disease who had visited the flower show or their relatives. The study was approved by the medical ethical committee of the Academic Medical Center in Amsterdam.

The following definitions were used to categorize the patients:“confirmed Legionnaires’ disease” was defined as the presence of a new infiltrate shown on the chest x-ray on admission and one or more of the following laboratory criteria: 1) isolation of L. pneumophila from a respiratory sample (28 patients), 2) detection of L. pneumophila serogroup 1 antigen in a urine sample (Binax Now Legionella urinary antigen test; Binax, Portland, ME) (86 patients), 3) seroconversion to positive immunoglobulin (Ig)G or IgM (or both) antibody levels to L. pneumophila, or a fourfold rise in antibody titers to L. pneumophila in paired acute-phase and convalescent-phase sera (62 patients). Antibodies to L. pneumophila were determined by using a commercial enzyme linked immunosorbent assay (IgM cutoff >140 U/ml and IgG cutoff > 70 U/ml, serogroup 1–7; Serion, Institut Virion-serion GmbH, Wurzburg, Germany) or a microagglutination antibody assay (IgM, serogroup 1-12; Regional Laboratory of Public Health, Tilburg, the Netherlands). In three patients, a commercial enzyme immunoassay (Binax EIA, Binax; Biotest EIA, Biotest AG, Dreiech, Germany) was positive in concentrated urine, while other diagnostic tests were negative.

“Probable Legionnaires’ disease” was defined as the presence of a new infiltrate shown on the chest x-ray on admission and either a single high antibody titer (microagglutination assay 1:>256; 2 patients) or a positive polymerase chain reaction analysis of sputum (1 patient) (10). Patients who visited the flower show and in whom radiologically confirmed pneumonia developed within 4 weeks were also considered to have probable Legionnaires’ disease when no other cause of the pneumonia could be established (18 patients). Patients were excluded if the first symptoms occurred more than 4 weeks after they visited the flower show. None of the patients were hospitalized during the month preceding admission.

### Data Collection and Definitions

Data on the following variables were collected from the medical chart (if data were missing, patients were interviewed by telephone): ,1) Premorbid conditions: age, sex, smoking >1 cigarette per day), alcohol intake (>2 units per day), use of immunosuppressive medication (ongoing treatment with chemotherapy or steroids >10 mg/day), underlying diseases such as chronic obstructive pulmonary disease, diabetes mellitus, chronic renal insufficiency, cancer (solid or hematologic neoplasm), and chronic cardiac disease (considered present if cardiac medication was used at the time of the flower show visit). 2) Day of visiting the flower show, first day of illness, and date of admission 3) Symptoms and results of physical examination on admission. 4) Routine biochemical and hematologic laboratory tests obtained on admission. 4) Urinary antigen test results collected from the microbiologic laboratory that performed the test. The overall agreement between the Binax NOW and the enzyme immunoassay Binax EIA (Binax Legionella Urinary Antigen EIA Kit: Binax) has been found to be 98% (11). 6) Chest radiograph results on admission and 48–72 hours later reviewed by attending hospital radiologist. Radiographic progression during this period was defined as an increase in density or size of infiltrate, or progression to multiple lobes. 7) Antibiotic treatment. Adequate therapy was considered a macrolide or a fluoroquinolone, with or without rifampicin. 8) Admission to the ICU, death, and renal insufficiency.

At the time of the patient’s admission, Legionnaires’ disease was defined as severe when two or more of the following conditions were present: 1) respiratory rate >30 breaths /minute, 2) chest radiograph showing bilateral involvement or involvement of multiple lobes, 3) shock (systolic blood pressure below 90 mmHg or diastolic blood pressure below 60 mmHg), 4) PaO2 <60 mmHg or arterial oxygen saturation <92%. For assessment of severity, we used the minor criteria for severity of community-acquired pneumonia described by the American Thoracic Society (12) since the major criteria are indicators for ICU admission by themselves.

### Statistical Analysis

The independent relation between clinical factors and the dependent variable, ICU admission or death (whatever came first), were assessed with univariate and multivariate logistic-regression models. Factors with a p value >0.20 in the multivariate analysis were excluded from the final multivariate analysis. Continuous variables were compared using a t test for groups; categorical variables were compared by using the chi-square test. A two-tailed p value of 0.05 or less was considered to indicate statistical significance.

Kaplan Meier survival analysis was used to compare the ICU-free survival between patients in whom adequate therapy was initiated within or later than 24 hours after admission. ICU-free survival was defined as survival without admission to the ICU during hospitalization.

## Results

### Patients

**Figure 1:**
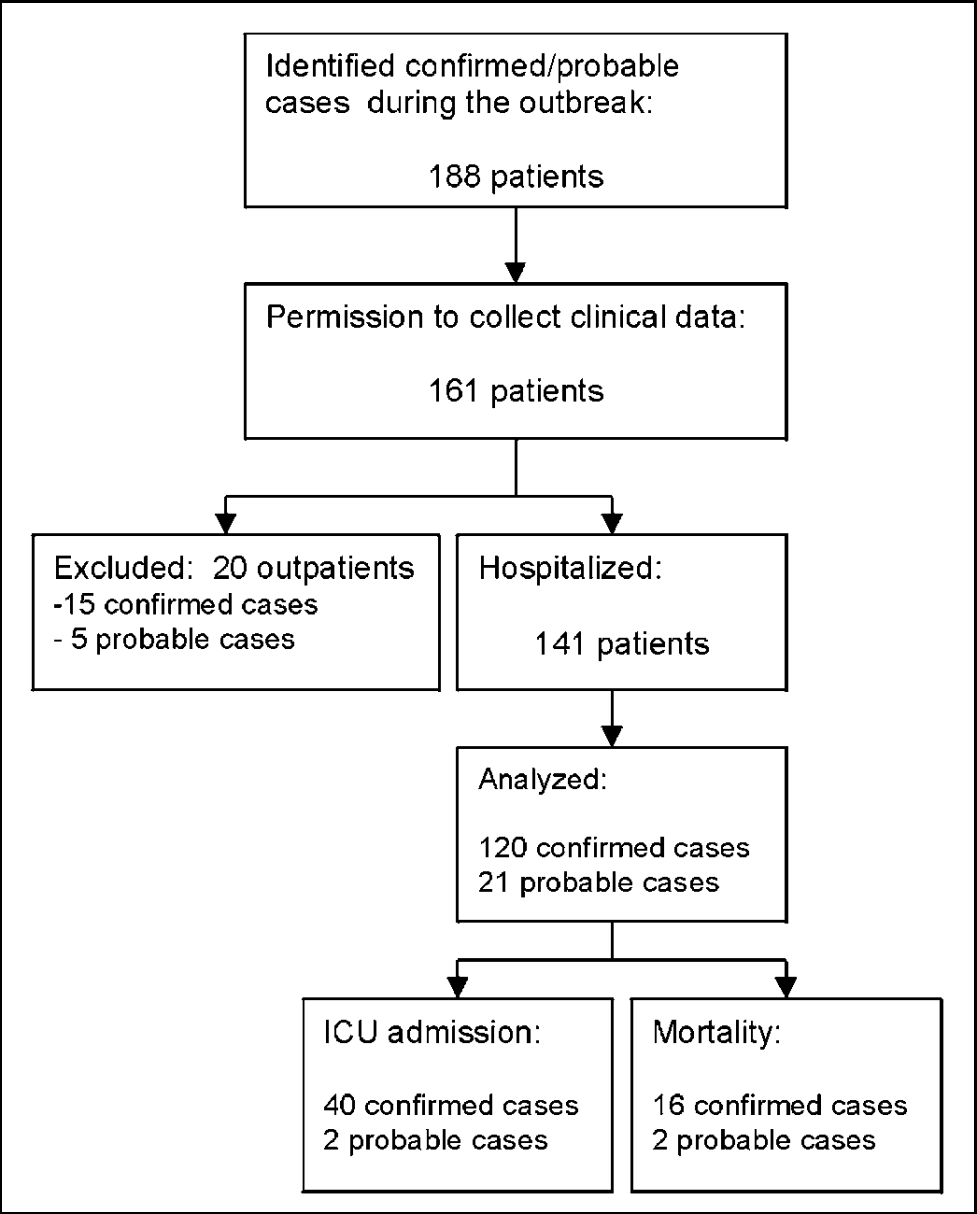
Patient disposition and selection. ICU, intensive care unit.

Of 188 identified patients with confirmed or probable Legionnaires’ disease during the outbreak (8), 161 patients gave permission to collect clinical data (Figure 1). Since severity of illness did not warrant hospital admission in 20 patients and limited clinical, laboratory and radiologic information was available for these 20 patients, they were not included in the final analysis. Among these 20 patients were 15 confirmed and 5 probable cases; none of these patients died during the course of Legionnaires’ disease. The final analysis was done on 141 hospitalized patients.

Forty-two (30%) of these 141 patients were admitted to the ICU, 40 (95%) of whom had confirmed Legionnaires’ disease. Overall mortality rate was 13%, and ICU mortality rate was 36% (Figure 1). The median incubation time was 7 days (range 1–18 days). The incubation time did not significantly differ between patients with severe Legionnaires’ disease (mean 6.8 days, [SD 3.5]) and those with nonsevere pneumonia (mean 7.8 days [SD 3.1]: t test p=0.13) or between patients who were admitted to the ICU or died (mean 7.0 days, [SD 3.1]) and those who did not (mean 7.7 days, [SD 3.5]: t test p=0.26).

The nationwide alert on March 12 led to an increase in hospital admissions. Patients admitted after the alert (n=71) were less severely ill: 21% had severe Legionnaires’disease in contrast to 44% before the alert (n=70). As expected, patients with severe Legionnaires’ disease (46/141, 33%) had an increased risk for ICU admission or death compared with nonseverely ill patients (OR 4.5, CI 2.1 to 9.6), p=0.001).

### Characteristics on Admission

The patients’ clinical, laboratory, and radiologic data on admission are shown in Table 1. The median age was 67 years (range 21–92 years) and more were male (58%). Eighty-eight (62%) patients had at least one underlying disease; cardiac disease was the most common. In the univariate analysis, smoking, dyspnea, fever above 38.5°C, plasma creatinine level >100 μmol/L, and bilateral infiltrates or pleural effusions shown on the chest x-ray at admission were found to predict subsequent ICU admission or death (Table 1). In the multivariate analysis, smoking, temperature >38.5°C, and bilateral infiltrates on admission were independent risk factors for ICU admission or death (Table 2).

**Table 1:** Univariate analysis of factors determining outcome^a^

	No. patients (%)	Odds ratio (95% CI)^b^	p value
Patient characteristics			
Male	82 (58)	1.5 (0.7 to 3.1)	0.30
Age >67 years	75 (53)	1.0 (0.5 to 2.1)	0.98
Underlying diseases			
COPD	11 (8)	0.8 (0.2 to 3.1)	0.73
Diabetes mellitus	16 (11)	1.0 (0.3 to 3.0)	0.95
Renal insufficiency	3 (2)	1.1 (0.1 to 12.1)	0.96
Cardiac disease	48 (34)	1.0 (0.5 to 2.0)	0.90
Cancer	10 (7)	0.5 (0.1 to 2.5)	0.41
Immunosuppressive medication^d^	11 (8)	1.9 (0.5 to 6.5)	0.52
Smoking^c^	65 (48)	2.4 (1.2 to 5.1)	0.02
Alcohol intake^c^	26 (59)	3.7 (0.8 to 15.8)	0.08
Symptoms			
Fever	119 (84)	0.8 (0.3 to 2.0)	0.63
Myalgia	31 (22)	0.6 (0.2 to 1.4)	0.21
Headache	36 (26)	0.6 (0.3 to 1.5)	0.30
Cough	97 (69)	1.0 (0.5 to 2.2)	0.99
Dyspnea	79 (56)	2.6 (1.2 to 5.5)	0.01
Diarrhea	25 (18)	1.3 (0.5 to 3.1)	0.63
Confusion	31 (22)	1.8 (0.8 to 4.0)	0.18
Physical examination			
Temperature >38.5°C	101 (72)	3.6 (1.4 to 9.3)	0.009
Respiratory rate >18/minc	34 (85)	5.6 (0.6 to 53.4)	0
Biochemistryc			
Sodium <130 mmol/L	36 (26)	2.1 (1.1 to 4.7)	0.06
Creatinine >100 µmol/L	73 (52)	2.1 (1.0 to 4.4)	0.05
CPK >200 U/L	25 (50)	1.4 (0.5 to 4.2)	0.57
ASAT >100 U/L	21 (18)	1.7 (0.6 to 4.4)	0.30
γ-GT >100 U/L	12 (13)	0.42 (0.09 to 2.03)	0.28
PO2 <9.7 kPa	96 (83)	0.64 (0.24 to 1.70)	0.37
X-ray results			
Bilateral infiltrates^e^	38 (27)	3.5 (1.6 to 7.6)	0.002
Pleural effusion	15 (11)	3.8 (1.2 to 11.3)	0.002
Progression within 48 hrsc,^f^	46 (41)	1.6 (0.7 to 3.4)	0.25

aCI: confidence interval, COPD, chronic obstructive pulmonary disease, CPK, creatinine phosphokinase; ASAT, aspartate aminotransferase; γ-GT, gamma glutamyltransferase. bLogistic regression analysis. cData for smoking (≥1 cigarette per day), alcohol intake (≥2 U per day), breathing frequencies, laboratory tests, and progression of infiltrates were available for a proportion of patients. Cutoff levels for CPK, ASAT, and γ-GT are two times the upper normal limit. dImmunosuppressive medication is defined as ongoing treatment with chemotherapy or steroids >10 mg/day. eUnilateral infiltrate was the reference group. f Radiographic progression during 24–48 hours was defined as an increase in density or size of the infiltrate, or progression to multiple lobes.

**Table 2:** Multivariate analysis of factors determining outcome

Prognostic factor^a^	Odds ratio (95% CI)^b^ (n=141)	p value
Smoking	2.5 (1.1 to 5.6)	0.03
Dyspnea at presentation	2.1 (0.9 to 4.8)	0.09
Temperature >38.5°C	2.9 (1.0 to 8.6)	0.05
Plasma creatinine >100 µmol/L	2.0 (0.9 to 4.6)	0.11
Bilateral infiltrates	4.2 (1.7 to 10.3)	0.002
Pleural effusion	3.4 (0.99 to 11.6)	0.053

aPrognostic factors with p≤ 0.05 in the univariate analysis were entered. Factors with p value >0.2 in the multivariate analysis were excluded. bCI, confidence interval.

During hospitalization, lung infiltrates shown on the chest x-ray progressed within 24–48 hours in 40% of the patients. This progression was not associated with ICU admission or death. In 39 patients (35%), renal insufficiency developed during admission (serum creatinine level above 130 μmol/L at any time during admission). Development of renal insufficiency was associated with ICU admission or death (OR 5.4, CI 2.3 to 12.7). None of the patients who survived had persistent renal insufficiency. In this large group of patients with Legionnaires’ disease, no other symptoms suggested extrapulmonary foci of infection.

### Therapy and Delay in Therapy

**Figure 2:**
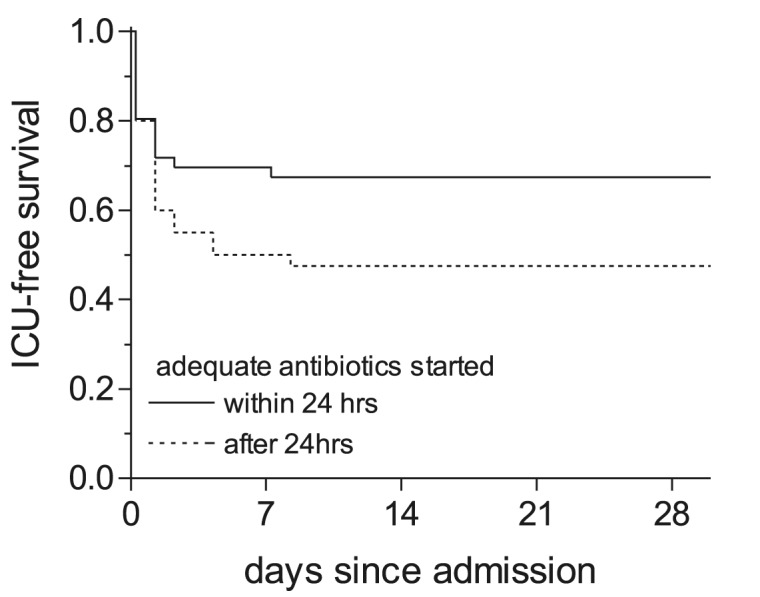
Kaplan-Meier curve for intensive care unit (ICU)–free survival. ICU-free survival for patients treated with adequate antibiotics within and >24 h after admission.:___ adequate antibiotic therapy started within 24 h after admission (n=85); ----- adequate antibiotic therapy started >24 h after admission (n=56).

Of the 70 patients admitted before the nationwide alert on March 12, 44 (63%) were treated with adequate antibiotics with a median delay of 1.5 days (range 0–14 days). After the alert, antibiotics were changed to a macrolide or a fluoroquinolone for 21 patients, and 5 patients were never treated with adequate antibiotics (three of them died). All patients admitted after the alert received adequate therapy within a median of 0 days (range 0–3 days). Next, we studied the influence of immediate start of adequate treatment compared with delayed treatment on the outcome. Initiation of adequate therapy within 24 hours after admission resulted in a higher ICU-free survival rate compared with initiation after 24 hours: 78% versus 54% (Figure 2; log rank: p= 0.005). The difference in ICU-free survival was not explained by differences in severity of pneumonia in the two groups, since the percentage of patients with severe pneumonia in the group treated within 24 hours (31% severe Legionnaires’ disease) did not significantly differ from the percentage in the group adequately treated after 24 hours (36% severe Legionnaires’ disease; chi square: p=0.5).

**Figure 3:**
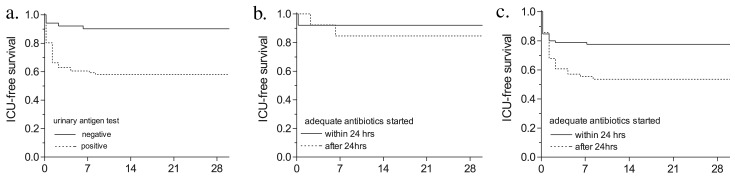
Survival curves and urinary antigen test results. A: Intensive care unit ICU)–free survival for patients with a positive or negative urinary Legionella antigen test (Binax Now, Binax, Portland, ME):___ negative urinary antigen test (n=51); ----- positive urinary antigen test (n=86). B: ICU-free survival for patients with a negative urinary Legionella antigen test (Binax Now):___ adequate antibiotic therapy started within 24 h after admission (n=38); ----- adequate antibiotic therapy started more than 24 h after admission (n=13). C: ICU-free survival for patients with a positive urinary Legionella antigen test (Binax Now): ___ adequate antibiotic therapy started within 24 h after admission (n=.46); ----- adequate antibiotic therapy started >24 h after admission (n=40).

A Binax Now urinary antigen test with positive results can provide a diagnosis of Legionella pneumonia within 1 hour. This test was positive in 86/141 (61%), negative in 51/141 (36%) and not done in 4/141 (3%) of the patients. Table 3 shows the results of other diagnostic tests of patients with positive and negative urinary antigen test results. In 16 patients with negative urinary antigen test results, no other test had positive results, although the clinical and epidemiologic features strongly suggested Legionnaires’ disease. Two of these patients were admitted to the ICU. Patients with negative urinary antigen test results had a higher ICU-free survival rate than patients with positive test results: 90% ICU-free survival compared with 58% of those with positive test results (Figure 3A; log rank: p=0.0001). No effect on outcome was found when initiation of adequate therapy was delayed in patients with a negative urinary antigen test (Figure 3B, 92% vs. 84% ICU-free survival; log rank: p= 0.46). In contrast, patients with positive urinary antigen test results in whom adequate therapy was started within 24 hours after admission had a higher ICU-free survival rate compared with patients in whom therapy was initiated after 24 hours (67% vs. 48% , Figure 3C; log rank: p= 0.09), resulting in a relative risk reduction of 38%. The proportion of patients with severe pneumonia was comparable for both groups of patients with a positive urinary antigen test (within 24 hours: 39%, after 24 hours, 45% severe Legionnaires’ disease, chi square: p= 0.58).

**Table 3:** Positive results of other diagnostic tests of patients with positive and negative urinary antigen tests^a^

Diagnostic test	Positive urinary antigen testb (n=86)	Negative urinary antigen test (n=51)
Sputum culture	23	3
Fourfold rise in titer/seroconversion	35	27
Polymerase chain reaction	9	1
Single high titer	5	3
Positive urinary antigen ELISA^b^ test in concentrated urine^c^	NA	7
No positive test results	29	17

aNA, not applicable; ELISA, enzyme-linked immunosorbent assay. bUrinary antigen test results were based on the test result of the qualitative immunochromatographic assay (Binax Now, Binax, Portland, ME). cPositive Binax EIA and Biotest EIA in concentrated urine samples.

In addition, 36 (38%) of 95 patients with nonsevere Legionnaires’ disease were treated with adequate antibiotic therapy >24 hours after admission; 13 of those patients (36%) had a poor outcome. In 10 (77%) of these patients, the urinary antigen test was positive for L. pneumophila, indicating that these patients should have been identified as high risk on admission.

## Discussion

 Since the first outbreak of Legionnaires’ disease in Philadelphia in 1976 (7), several outbreaks have been described that were linked to hospitals, hotels, cooling-towers, and whirlpool baths (13–16). The outbreak reported here is the largest outbreak associated with a contaminated whirlpool spa located at the exhibition hall of a flower exhibition. Analysis of 141 hospitalized patients showed that a history of smoking, fever >38.5°C, and bilateral infiltrates shown on chest x-ray were associated with an increased risk for ICU admission or death. A urinary antigen test with positive results was also associated with poor outcome. Initiation of adequate therapy within 24 hours after admission showed a higher ICU-free survival rate compared to initiation of therapy after 24 hours. No protective effect of early adequate therapy was found in patients with Legionnaires’ disease and a urinary antigen test negative for L. pneumophila. However, in patients with positive urinary test results, early adequate therapy reduced the risk of ICU admission and death by 38%.

The endpoint of either ICU admission or death was chosen because only 18 patients died, which strongly decreased the power of the analysis. In clinical practice, preventing ICU admission with all the disadvantages of such an admission in terms of sickness and death, is one of the goals of early treatment. Since >80% of the diseased patients were first admitted to the ICU, we chose to combine ICU admission and death as a composite primary outcome parameter.

We analyzed 141 hospitalized patients and excluded 20 outpatients. However, this group represents only 20 out of 161 patients, and the described 141 patients represent 88% of all patients. In a study by Boshuizen et al. (17), a survey among the 700 exhibitors at the flower show revealed no symptomatic infections (for example, Pontiac fever); these researachers concluded that either pneumonia develops in exposed persons or they remain healthy. Therefore, this study elucidates the complete range of the severity of the pneumonia that develops in these patients.

The case definition for probable cases was broad enough to ensure inclusion of patients who died before the diagnostic work-up for Legionella was completed. Of the 21 probable case-patients, 18 had no single diagnostic test with positive results and showed no evidence of infection by other microorganisms (4 ICU admissions of which 2 died). Despite clinical and epidemiologic features suggestive of Legionnaires’ disease, other undetected causes of pneumonia cannot be excluded.

Patients with Legionnaires’ disease are more likely to have severe pneumonia requiring ICU admission than are patients with community-acquired pneumonia caused by other organisms (1,18,19). In this study, 42 (30%) patients) were admitted to the ICU. The overall mortality rate (13%) and ICU mortality rate (36%) in our patients were consistent with earlier reports (1,3,4).

For all patients, the exposure day and the date when first symptoms occurred were known. The incubation time ranged from 1 to 18 days, which is longer than the upper limit of 12 days reported previously (7,20). The virulence of the causative Legionella strain, as assessed by its potential for intracellular growth (21), did not differ from that of other clinical isolates and cannot account for this long incubation time. This longer incubation means that Legionnaires’ disease can no longer be excluded as a potential cause of community-acquired pneumonia when, for example, the person traveled >12 days ago.

Male gender, older age, underlying diseases like chronic obstructive pulmonary disease, diabetes mellitus, and immunosuppressive medication, reported by others as predictors for fatal outcome (2–4), were not associated with poor outcome in this study, although the prevalence of underlying diseases in our population was similar to the prevalence in earlier studies describing community-acquired Legionnaires’ disease. This difference from other studies might be explained by the fact that in our study no selection was made for ICU patients, and no patients with nosocomial disease were included (2–4,22).

Patients who sought treatment with bilateral infiltrates (27%) and with pleural effusion (11%) had an increased risk for ICU admission or death. In a prospective study on chest radiographic findings in patients with community-acquired Legionnaires’ disease, 16% of the patients had bilateral involvement, and 23% had pleural effusions on admission, which increased to 30% and 63%, respectively, during hospitalization (23). Despite some lung deterioration, which was visible on chest x-ray, most patients improved clinically. In this study, progressive lung deterioration within 48–72 hours (noted in 41% of the patients) was not a significant risk factor for ICU admission or death. Bilateral involvement on admission, on the other hand, was the most powerful prognostic factor associated with poor outcome in the multivariate analysis.

Identification of patients with Legionnaires’ disease has important implications for the choice of initial therapy. Studies comparing the clinical manifestations of Legionella pneumonia to other types of pneumonia have indicated that Legionnaires’ disease is not “atypical” and that individual clinical features such as diarrhea, confusion, hyponatremia, and chest x-ray findings are not sufficiently distinctive to distinguish Legionnaires’ disease from other types of community-acquired pneumonia (18,24–26). The results of cultures require several days, and serum antibody tests have a low sensitivity. In patients with Legionnaires’ disease related to this outbreak, sensitivity was approximately 43% for one of the three separate antibody tests and 61% for any of the tests, using a positive culture result, a urinary antigen test with positive results, or both as the criterion standard (E. Yzerman, pers. comm.).

Detection of L. pneumophila antigens in a urine sample provides a diagnosis within 1 hour, with a specificity of 95% to 100% (11) (Binax Now Legionella urinary antigen test, Binax). Patients in our study with a positive urine test during hospitalization had an increased risk for ICU admission or death, in accordance with data indicating that the percentage of positive test results increased with the clinical severity of the disease (27). Although the urinary antigen test was done retrospectively in many patients (median 9 days after the first symptoms, range 0–25 days), the number of positive urinary tests is not lower during the first 3 days after symptoms than after 3 weeks of illness (28). The urinary antigen test used during this outbreak detects L. pneumophila serogroup 1, which is responsible for approximately 70%–80% of Legionnaires’ disease cases in the United States and Europe.

Increased deaths associated with delay of adequate treatment for Legionnaires’ disease has been reported earlier (1,5); in patients suspected of having Legionnaires’ disease, adequate therapy should therefore be started as soon as possible. To ensure coverage of potential L. pneumophila infections in every patient, the recommendations for treatment of patients with community-acquired pneumonia have been expanded. The new guidelines from the Infectious Diseases Society of America recommend an extended-spectrum cephalosporin plus a macrolide or a fluoroquinolone alone, for every hospitalized patient in whom no pathogen is defined (6). This approach may lead to overtreatment since 2%–13% of community-acquired pneumonia is caused by L. pneumophila (18,29,30). Therefore, this approach is costly and, in addition, may contribute to macrolide and fluoroquinolone resistance.

The results of our study suggest that a more tailored approach of patients with community-acquired pneumonia may be possible. When Legionnaires’ disease is considered in the differential diagnosis of patients with community-acquired pneumonia, a urinary antigen test should be done on admission. If test results are positive, the patient should be treated immediately with a fluoroquinolone or a macrolide since a positive urinary test on admission identifies the patients with Legionnaires’ disease caused by L. pneumophila serogroup 1 and a high risk for ICU admission or death. If the urinary antigen test gives negative results, deferring anti-Legionella therapy for the first 24 hours after admission, pending the diagnostic work-up, may be justified because the outcome in Legionnaires’ disease is not influenced. In this way, unnecessary use of antibiotics in patients hospitalized with community- acquired pneumonia may be avoided.

## References

[1] Falco V. Fernandez de Silva, Alegre J, Ferrer A, Martinez Vasguez JM. Legionella pneumophila: a cause of severe community-acquired pneumonia. Chest. 1991;100:1007–11. 10.1378/chest.100.4.1007 1914547

[2] Marston BJ, Lipman HB, Breiman RF. Surveillance for Legionnaires’ disease: risk factors for morbidity and mortality. Arch Intern Med. 1994;154:2417–22. 10.1001/archinte.154.21.2417 7979837

[3] el-Ebiary M, Sarmiento X, Torres A, Nogue S, Mesalles E, Bodi M, Prognostic factors of severe Legionella pneumonia requiring admission to ICU. Am J Respir Crit Care Med. 1997;156:1467–72. 9372662 10.1164/ajrccm.156.5.97-04039

[4] England AC, Fraser DW, Plikaytis BD, Tsai TF, Storch G, Broome CV. Sporadic legionellosis in the United States: the first thousand cases. Ann Intern Med. 1981;94:164–70. 7469207 10.7326/0003-4819-94-2-164

[5] Heath CH, Grove DI, Looke DF. Delay in appropriate therapy of Legionella pneumonia associated with increased mortality. Eur J Clin Microbiol Infect Dis. 1996;15:286–90. 10.1007/BF01695659 8781878

[6] Bartlett JG, Dowell SF, Mandell LA, File TM Jr, Musher DM, Fine MJ. Practice guidelines for the management of community-acquired pneumonia in adults. Clin Infect Dis. 2000;31:347–82. 10.1086/313954 10987697 PMC7109923

[7] Fraser DW, Tsai TR, Orenstein W, Parkin WE, Beecham HJ, Sharrar RG, Legionnaires’ disease: description of an epidemic of pneumonia. N Engl J Med. 1977;297:1189–97. 335244 10.1056/NEJM197712012972201

[8] Den Boer JW, Yzerman EP, Schellekens JFP, Lettinga KD, Boshuizen HC, van Steenbergen JE, A large outbreak of Legionnaires’ disease at a Dutch flower show. Emerg Infect Dis. 2002;1:37–43. 10.3201/eid0801.010176 PMC2730281 11749746

[9] Steenbergen JE, Slijkerman FAN, Speelman P. The first 48 hours of investigation and intervention of an outbreak of legionellosis in the Netherlands. Eurosurveillance. 1999;4:112–5. 10.2807/esm.04.11.00059-en 12631882

[10] Zee van der A, Verbakel H, Jong de C, Pot R, Peeters M, Schellekens J, . A clinical validation of diagnosis of Legionella infections. In: Abstracts of the 5th international conference on Legionella; Ulm, Germany; 2000 Sept 26–29; Abstract 49. Washington, D.C.: American Society for Microbiology; 2001.

[11] Dominguez J, Gali N, Matas L, Pedroso P, Hernandez A, Padilla E, Evaluation of a rapid immunochromatographic assay for the detection of Legionella antigen in urine samples. Eur J Clin Microbiol Infect Dis. 1999;18:896–8. 10.1007/s100960050427 10691203

[12] Niederman MS, Mandell LA, Anzueto A, Bass JB, Broughton WA, Campbell GD, Guidelines for the management of adults with community-acquired pneumonia: diagnosis, assessment of severity, antimicrobial therapy, and prevention. Am J Respir Crit Care Med. 2001;163:1730–54. 11401897 10.1164/ajrccm.163.7.at1010

[13] Kool JL, Fiore AE, Kioski CM, Brown EW, Benson RF, Pruckler JM, More than 10 years of unrecognized nosocomial transmission of Legionnaires’ disease among transplant patients. Infect Control Hosp Epidemiol. 1998;19:898–904. 9872525 10.1086/647760

[14] Bell JC, Jorm LR, Williamson M, Shaw NH, Kazandjian DL, Chiew R, Legionellosis linked with a hotel car park—how many were infected? Epidemiol Infect. 1996;116:185–92. 10.1017/S0950268800052420 8620910 PMC2271622

[15] Dondero TJJ, Rendtorff RC, Mallison GF, Weeks RM, Levy JS, Wong EW, An outbreak of Legionnaires’ disease associated with a contaminated air-conditioning cooling tower. N Engl J Med. 1980;302:365–70. 7351928 10.1056/NEJM198002143020703

[16] Jernigan DB, Hofmann J, Cetron MS, Genese CA, Nuorti JP, Fields BS, Outbreak of Legionnaires’ disease among cruise ship passengers exposed to a contaminated whirlpool spa. Lancet. 1996;347:494–9. 10.1016/S0140-6736(96)91137-X 8596266

[17] Boshuizen HC, Neppelenbroek SE, Van Vliet H, Schellekens JFP, Den Boer JW, Peeters MF, Subclinical Legionella infection in workers near the source of a large outbreak of Legionnaires’ disease. J Infect Dis. 2001;184:515–8. 10.1086/322049 11471112

[18] Fang GD, Fine M, Orloff J, Arisumi D, Yu VL, Kapoor W, New and emerging etiologies for community-acquired pneumonia with implications for therapy: a prospective multicenter study of 359 cases. Medicine. 1990;69:307–16. 10.1097/00005792-199009000-00004 2205784

[19] Pedro-Botet ML, Sabria-Leal M, Haro M, Rubio C, Gimenez G, Sopena N, Nosocomial and community-acquired Legionella pneumonia: clinical comparative analysis. Eur Respir J. 1995;8:1929–33. 10.1183/09031936.95.08111929 8620964

[20] Hoge CW, Brieman RF. Advances in the epidemiology and control of Legionella infections. Epidemiol Rev. 1991;13:329–40. 1765117 10.1093/oxfordjournals.epirev.a036076

[21] Neumeister B, Schoniger S, Faigle M, Eichner M, Dietz K. Multiplication of different Legionella species in Mono Mac 6 cells and in Acanthamoeba castellanii. Appl Environ Microbiol. 1997;63:1219–24. 9097418 10.1128/aem.63.4.1219-1224.1997 PMC168415

[22] Almirall J, Mesalles E, Klamburg J, Parra O, Agudo A. Prognostic factors of pneumonia requiring admission to the intensive care unit. Chest. 1995;107:511–6. 10.1378/chest.107.2.511 7842786

[23] Tan MJ, Tan JS, Hamor RH, File TMJ, Breiman RF. the Ohio Community-Based Pneumonia Incidence Study Group. The radiologic manifestations of Legionnaires’ disease. Chest. 2000;117:398–403. 10.1378/chest.117.2.398 10669681

[24] Torres A, Serra-Batlles J, Ferrer A, Jimenez P, Celis R, Cobo E, Severe community-acquired pneumonia: epidemiology and prognostic factors. Am Rev Respir Dis. 1991;144:312–8. 1859053 10.1164/ajrccm/144.2.312

[25] Sopena N, Sabria-Leal M, Pedro-Botet ML, Padilla E, Dominguez J, Morera J, Comparative study of the clinical presentation of Legionella pneumonia and other community-acquired pneumonias. Chest. 1998;113:1195–200. 10.1378/chest.113.5.1195 9596294

[26] Lieberman D, Porath A, Schlaeffer F, Boldur I. Legionella species community-acquired pneumonia: a review of 56 hospitalized adult patients. Chest. 1996;109:1243–9. 10.1378/chest.109.5.1243 8625675

[27] Wever PC, Yzerman EP, Kuijper EJ, Speelman P, Dankert J. Rapid diagnosis of Legionnaires’ disease using an immunochromatographic assay for Legionella pneumophila serogroup 1 antigen in urine during an outbreak in the Netherlands. J Clin Microbiol. 2000;38:2738–9. 10878074 10.1128/jcm.38.7.2738-2739.2000 PMC87013

[28] Kohler RB, Winn WCJ, Wheat LJ. Onset and duration of urinary antigen excretion in Legionnaires’ disease. J Clin Microbiol. 1984;20:605–7. 6490846 10.1128/jcm.20.4.605-607.1984 PMC271393

[29] Sopena N, Sabria M, Pedro-Botet ML, Manterola JM, Matas L, Dominguez JA, Prospective study of community-acquired pneumonia of bacterial etiology in adults. Eur J Clin Microbiol Infect Dis. 1999;18:852–8. 10.1007/s100960050419 10691195

[30] Marston BJ, Plouffe JF, File TMJ, Hackman BA, Salstrom SJ, Lipman HB, Incidence of community-acquired pneumonia requiring hospitalization: results of a population-based active surveillance. Arch Intern Med. 1997;157:1709–8. 10.1001/archinte.157.15.1709 9250232

